# Significance of chronic toxoplasmosis in epidemiology of road traffic accidents in Russian Federation

**DOI:** 10.1371/journal.pone.0184930

**Published:** 2017-09-28

**Authors:** Ekaterina V. Stepanova, Anatoly V. Kondrashin, Vladimir P. Sergiev, Lola F. Morozova, Natalia A. Turbabina, Maria S. Maksimova, Alexey I. Brazhnikov, Sergei B. Shevchenko, Evgeny N. Morozov

**Affiliations:** 1 Martsinovsky Institute of Medical Parasitology, Tropical and Vector Borne Diseases, I.M. Sechenov First Moscow State Medical University, Moscow, Russian Federation; 2 Department of Tropical Medicine and Parasitic Diseases, I.M. Sechenov First Moscow State Medical University, Moscow, Russian Federation; 3 Department of Epidemiology and Evidence-based Medicine, I.M. Sechenov First Moscow State Medical University, Moscow, Russian Federation; 4 Department of Tropical, Parasitic Diseases and Disinfectology, Russian Medical Academy of Continuous Professional Education, Moscow, Russian Federation; Universita degli Studi di Parma, ITALY

## Abstract

Studies carried out in Moscow residents have revealed that the prevalence of chronic toxoplasmosis is very close to those in countries of Eastern and Central Europe. Our findings also demonstrated a statistically significant relationship between the rate of traffic accidents and the seroprevalence of chronic toxoplasmosis in drivers who were held responsible for accidents. The latter was 2.37 times higher in drivers who were involved in road accidents compared with control groups. These results suggest that the consequences of chronic toxoplasmosis (particularly a slower reaction time and decreased concentration) might contribute to the peculiarities of the epidemiology of road traffic accidents in the Russian Federation and might interfere with the successful implementation of the Federal Programme named “Increase road traffic safety”. Suggestions for how to address overcome this problem are discussed in this paper.

## Introduction

Road traffic accidents (RTAs) are very serious health and social problems worldwide. In the Russian Federation, this problem is particularly acute compared with other countries that have similar levels of social and economic development. According to the WHO Road Safety Estimations, the road traffic death rate (per 100000 population) in the Russian Federation was 18.9 in 2013, which was two times lower than that in many developing countries [1] but much higher compared with European countries such as the Netherlands (3.4), Germany (4.3), Finland (4.8), and the Czech Republic (6.1) [2].

The demographic burden of RTA and their consequences is enormous. According to the State Statistical Bureau of Russia, during the period 1985–2012, the total number of RTAs in the Russian Federation exceeded 5 million, with more than 850000 people dead. More than 6 million suffered injuries of various degrees of severity. Major trauma, including concomitant injuries, multi-organ injuries, and composite fractures, represents more than 60% of all injuries and leads to the handicap of more than 6000 persons annually. Reports from the Ministry of Health reveal that the total number of fatal cases due to RTAs is 12 times higher and that the number of disabilities is 6–7 times higher compared with that among other causes of injury. The annual economic burden due to the RTA in the Russian Federation is estimated at approximately 1 trillion rubles (US $ 16.7 billion as of 20.12.2016).

The government of the Russian Federation considers road safety to be one of the most important goals for social and economic development. An appreciation and concern for the magnitude of the problem is reflected in the allocation of considerable amount of funds to establish and implement various activities within the framework of the Federal Program “Increase road traffic safety, 2013–2020” under the lead agency Road Safety Commission of the Government of Russian Federation. The aim of the program, at the cost of almost 36 trillion rubles (US $36 billion), is the annual reduction of the number of fatal cases due to RTA by 29% (not more than 8000 cases) by 2020 compared to 2012. Prior to launching the Program, the epidemiological peculiarities of RTAs in the Russian Federation were ascertained and appear to be determined by interaction of several factors. One of the most important factors was believed to be the discordance between the ever-increasing intensity of road traffic (particularly for the last 15–20 years), followed by the tempo of the construction of new roads and the proper maintenance of the existing road network. It was also found that the violation of the National rules of the road was the cause of almost 85% of all RTAs, of which drivers were responsible for 70%-75% [3].

For 55% of the RTAs, the cause was exceeding the speed limit and breaking the rules at the regulated intersections (30%). A considerable number of road accidents have been committed by drivers under the influence of alcohol or drugs. Road traffic deaths involving alcohol represent 8% of all deaths on the roads [4]. However, one of the factors contributing to the peculiarities of the epidemiology of RTAs in Russia was not considered. This factor is the consequences of chronic toxoplasmosis among persons involved in RTAs. This was not analyzed, although a number of publications on the subject have been available abroad since the mid-**1990s** [5, 6].

Unlike its acute form, chronic toxoplasmosis is not accompanied by manifested clinical symptoms of the disease [7]. The chronic form of toxoplasmosis in the Russian Federation is still considered a phenomenon of asymptomatic carriage, and clinicians do not consider it a health problem. A previous study revealed that the prevalence of latent toxoplasmosis in the Russian Federation is 5%-7% in Republic Saha and Omsk province, both of which are in Eastern Siberia [8]. Investigations in other parts of the country have not been carried out.

Worldwide interest and concern for the emerging problem of toxoplasmosis, especially its chronic form, have been demonstrated over the last 15–20 years, when new dimensions of the disease were established. The results of monitoring *Toxoplasma*-infected persons have revealed behavioral changes among them compared with uninfected persons. The intensity of such changes is closely correlated with the duration of chronic toxoplasmosis [6,9,10]. It appears that the mechanism that determines the personality changes is associated with an increase in the production of the neurotransmitter dopamine, affecting a person’s motor activity, aggression and social behavior [11, 12]. Once in the nerve or inside the muscle tissue, the parasite forms cysts that cause the development of chronic toxoplasmosis [13,14]. It was shown that *Toxoplasm*a localizes in the nerve cells of the brain and stimulates the production of dopamine. This effect could be responsible for prolongation of person’s reaction time and ability to concentrate [15] and also for an increased risk of traffic and working place incidents, as convincingly demonstrated in epidemiological studies in the Czech Republic, the Republic of Turkey, and Mexico [16,17,18,19]. Subsequent observations among toxoplasmosis-infected persons conducted in various countries have confirmed significant behavioral changes, including personality changes, IQ loss and altered psychomotor activity, including an increased risk of involvement in road traffic accidents [10, 11,13].

Thus, the objectives of our study were a) to determine the prevalence of chronic toxoplasmosis in the city and region of Moscow and b) to establish the probable role of the disease in the epidemiology of RTAs in the Russian Federation to facilitate the successful implementation of the Federal Program.

## Methods

The study was performed at the Sechenov First Moscow State Medical University and the Moscow Sklifosovskii Institute of Emergency Medicine during 2015.

To establish the general prevalence of chronic toxoplasmosis among residents of Moscow city, examinations were carried out among persons attending the Outpatient Department at the Clinical Center of the Sechenov First Moscow State Medical University. No special criteria were selected for examination in terms of age, sex and occupation. A total of 1272 persons were examined (Table 1).

**Table 1:** Prevalence of chronic toxoplasmosis among the residents of Moscow city (Russian Federation), 2015.

Group	Examined	Positive results (ELISA test)	
		Absolute number	Percent (%)
Total number	1272	323	25.39
Men	497	120	24.14
Women	775	203	26.19

To address the second objective, we carried out an analytical epidemiological “case-control” study represented by two groups: experimental and control. The experimental group consisted of persons in possession of a valid driving license who were hospitalized because of a road traffic accident for which they were responsible. All persons in the experimental group were patients of the Sklifosovsky Medical Emergency Institute, Moscow. The criteria for inclusion were a) proof that the admitted person was driving at the time of the accident, b) evidence that their actions/behavior had led to the occurrence of the accident; and c) age from 18–45 years.

The criterion for exclusion was driving at the time of an accident under the influence of alcohol/drugs. A total of 100 persons constituted the experimental group, with 65 men and 35 women (Tables 2 and 3).

**Table 2:** Comparative prevalence of immunoglobulins M and G to *Toxoplasma gondii* in the experimental group and the control group.

Group	Examined	Absolute number		Positive results (%)	Odds ratio	C.I._95_	p-values
		IgM	IgG				
Case	100	0	45	45.00	2.37	1.34–4.2	0.001
Control	152	0	39	25.66			

**Table 3:** Comparative gender prevalence of chronic toxoplasmosis in the experimental group and the control group.

Group	Experimental group			Control group			Odds ratio	C.I._95_	p-values
	Examined	Positive results		Examined	Positive results				
		Absolute number	Percent (%)		Absolute number	Percent (%)			
Men	65	29	44.61	82	22	26.83	2.2	1.04–4.66	0.02
Women	35	16	45.71	70	17	24.28	2.63	1.02–6.8	0.02

The control group consisted of 152 healthy persons aged 18–45 years (82 men and 70 women), who were undergoing routine medical examinations at the Clinical Centre of Sechenov University.

Participants in the experimental and control groups were informed about the purposes of the study, and informed consent was obtained before enrollment in the study.

All study patients were tested for the presence of IgG- and IgM-specific antibodies to *Toxoplasma gondii*. The determination of specific immunoglobulin G and M in the blood serum of the study groups (experimental and control) were determined using “Vector-Toxo of IgG” (Vector-Best, Novosibirsk, Russian Federation) and “Vector Toxo-IgM” enzyme-linked immunosorbent assay (ELISA) test kits, produced by JSC “VECTOR-BEST.” The indicator of chronic toxoplasmosis in a patient was the presence of IgG in the absence of IgM [13].

The statistical significance of the results in the experimental and control groups was obtained using the χ² criterion, and the odds ratio (OR) was calculated with a level of reliability not less than 95%. The Statistical Package EpiInfo Version was employed for calculations. Additionally, we used the Pearson Correlation, Partial Correlation, and Mantel-Haenszel Common Odds Ratio Estimate.

### Ethical considerations

The study was approved by the Research Ethics Board of Health of the Sechenov First Moscow State Medical University (protocol № 04–13, 10.04.2013). Participants in the experimental and control groups were informed about the purposes of the study, and informed consent was obtained before enrollment in the study in verbal form.

## Results and discussion

The prevalence of chronic toxoplasmosis in the residents of Moscow city is presented in Table 1. These values were considerably higher (25.39%, n = 1272) in our study than those obtained earlier in Eastern Siberia in Russia [8]. This could be attributed to the differences in the living conditions and food preferences of the local population. However, our findings are quite close to the results obtained in Austria, Croatia, Slovenia, and Switzerland [20,21,22].

The results of the examination of blood serum in the experimental and control groups are presented in Tables 2 and 3.

As seen in Table 2, the immunoglobulin M was absent in both groups, whereas immunoglobulin G was present in 45% of those tested in the experimental group compared to 26% in the control group. The absence of the immunoglobulin IgM in conjunction with the presence of IgG suggests the presence of persons with exclusively the chronic form of toxoplasmosis in both the experimental and control groups. It was found that the number of seropositive subjects with IgG in the experimental group was significantly higher than that in the control group. Thus, among the persons involved in traffic accidents who were responsible for their occurrence, the incidence of cases with chronic toxoplasmosis was more than twice that in the control group.

The results of our studies are quite close to those obtained in the Czech Republic, where it was found that the risk for traffic accidents in subjects with chronic toxoplasmosis was 2.65 times (2.37 in our study) higher than in the control group [16].

In Turkey, which has a traffic toll of approximately 7500 persons annually, similar epidemiological studies revealed that the risk of accidents in the experimental group was between 2 and 4 times higher than that in the control group [17,18].

Thus, both our results and those obtained abroad suggest the presence of a link between the incidence of chronic toxoplasmosis and road traffic accidents.

The role of the consequences of chronic toxoplasmosis in the changes of the personality profile of an infected person has been well established. A slower reaction time and decreased concentration are most remarkable features among the changes in an infected person [6,23]. These changes are particularly important while driving [16]. The results of studies carried out among 3890 military drivers (men) in the Czech Republic revealed that persons with high titers of *Toxoplasm*a antibodies had a 6-fold higher frequency of accidents compared with uninfected persons [24].

It is believed that the mechanism of such phenomena in general is related to the modulation of dopamine levels (increased or decreased) as a result of the presence of the parasite in brain cells [25,26]. The consequences of such modulation can be seen in the changing behavior in affected individuals, with marked sex differences and similarities. Men with chronic toxoplasmosis have higher and women lower concentrations of testosterone [15]. The direction of toxoplasmosis-associated shift in intelligence, extroversion, suspiciousness, strength of superego, and self-sufficiency differ between men and women. However, no differences in the direction of such shifts were observed in consciousness, novelty seeking, and the reaction time impairment and these changes could result in a higher risk of road traffic accidents [5,6,14,27].

The data in Table 3 show that the effect of toxoplasmosis was significant and similar for men (OR = 2.2, CI_95_ = 1.04–4.66, p<0.02) and women (OR = 2.6, CI_95_ = 1.02–6.8,p<0.02). Similar prevalence of seropositive subjects was found in men and women and this was true both in the experimental and the control group despite the fact that the prevalence of toxoplasmosis was about two times higher in the experimental group.

To assess the possible confounding effects of sex, we performed also the Mantel-Haenzel test. The OR adjusted for sex was 2.35 CI_95_ 1.37–4.03.

It appears that similarities in the behavioral changes of infected men and women driving on the roads determines “aggressive driving,” the neglect of driving rules, and ignoring pedestrians. In conjunction with inadequate road conditions, which currently prevail in Russia, these factors might contribute to an increased risk of road traffic accidents.

As of today, the Federal Program is in its 4^th^ year of implementation (2013–2016). During that period, the proportion of the federal and regional network of roads that fully correspond to the national standards has increased from 39% in 2013 to 71% in 2016. However, an appreciable improvement of the road’s standards has not been accompanied by an expected reduction of the total number of RTA and its consequences, as exemplified by the following data. For example, in 2015, there were total of 151000 serious RTAs, with 19000 fatal cases. Approximately 190000 persons were injured with traumas of various degrees of severity. Over the first 9 months of 2016, the total reported number of RTAs was more than 133000, in which 16000 persons died and 168000 persons were injured. Thus, these data indicate that the “human factor”, drunk driving, a low rate of seat-belt wearing, excess speed, a violation of overtaking rules, inadequate behavior in extreme situations, not keeping a stipulated distance between vehicles while driving and the probable consequences of chronic toxoplasmosis—continue to play extremely important roles in the current epidemiology of RTAs in the Russian Federation. The limited scale of our studies did not allow us to precisely calculate the number of RTAs that were directly related to chronic toxoplasmosis. However, one cannot exclude the probability that the consequences of the disease might act in conjunction with other factors, for example, with the presence of alcohol in the blood of the person who caused the RTA. Another factor limiting the possibility to exactly quantify the real impacts of latent toxoplasmosis, e.g. to compute the attributable risk, is the fact that the results of several studies abroad have demonstrated that th Rh positive subjects, especially the heterozygotes, are protected against many negative effects of toxoplasmosis, including impairment of reaction times [28] and an increased risk of traffic accidents [23]. In addition, the impairment of reaction times increases [23,28] but the risk of traffic accidents decreases with the duration of the *Toxoplasma* infection [24].

We strongly feel that the results of our studies clearly indicate the necessity to address the consequences of chronic toxoplasmosis in the implementation of the Federal Program on Road Safety in Russian Federation, *on par* with other factors contributing to the problem of road accidents. One of the possible steps towards solving the problem might be an entry screening for toxoplasmosis in every student in every driving school. Licensed drivers should be tested for toxoplasmosis during routine medical examinations. Considering the magnitude of road accidents involving pedestrians, Special Programs should be developed to prevent road accidents, specifically programs targeting school children. To that effect, negotiations with the national authorities are already in progress. Thus, our experience could be extended to individuals who are engaged in other fields of activity, for example, the screening of conscripts and members of the military who drive and manage various military equipment, including aircraft.

It should be kept in mind, however, that the solution to chronic toxoplasmosis with respect to road safety lies in the development of efficient specific drugs targeting parasitic cysts.

### Strength and limitations of the study

The results of this study provide very useful information that can be used to educate physicians in the Russian Federation who are still unaware of the consequences of chronic toxoplasmosis. This information may also be used while assessing the progress of the Federal Program on road safety in Russia.

There are some confounders in our study that might have interfered with the reliability of the obtained results. This study is limited in terms of the generalizability of the findings, as it examined the inhabitants of Moscow city only. With respect to the overall prevalence of chronic toxoplasmosis among the residents of Moscow city, no data on the territorial distribution within the study area were ascertained. The availability of such data might provide an idea of whether the distribution of the infection is diffused or focal. With respect to the comparative prevalence of chronic toxoplasmosis in the experimental and control groups, the limiting factors could be considered to be as follows. First, the study was confined to drivers who were held responsible for RTAs. The role of pedestrians involved in RTAs and their responsibility were not studied. The combined prevalence in drivers and pedestrians might be higher. Second, the age groups were limited to only those aged 18–45 years. The inclusion of subject older than 45 years of age might increase or decrease the prevalence of chronic toxoplasmosis in both groups. Third, the possibility of different protection against negative effects of toxoplasmosis in RTAs among the Rh positive and Rh negative subjects was not established. Fourth, the decrease of the risk of traffic accidents during the *Toxoplasm*a infection was not studied.

## Conclusions

The results of our studies had revealed that frequency of chronic toxoplasmosis among residents of the city and region of Moscow in the Russian Federation is very close to those in many European countries. It was also demonstrated that the statistically significant frequency of chronic toxoplasmosis among drivers, regardless of gender and the responsibility for road accidents, was more than 2 times higher compared with that of the control group. The results of our studies are consistent with data from similar studies in other countries.

We suggest that the results of our research should be taken into consideration during the implementation of the Federal Road Safety Program. These results show that the effect of latent toxoplasmosis could play a very important role in the epidemiology of road accidents in the Russian Federation.

## Supporting information

S1 File Protocol. (PDF)
