# Supplementary material for: Significance of chronic toxoplasmosis in epidemiology of road traffic accidents in Russian Federation
Source: PLoS One. 2017 Sep 28;12(9):e0184930. doi: 10.1371/journal.pone.0184930 (PMC5619715; doi:10.1371/journal.pone.0184930)
Supplement: S1 File — (PDF) [file pone.0184930.s001.pdf]

Significance of chronic toxoplasmosis in epidemiology of road traffic accidents in Russian Federation

(Protocol of the investigation)

Objectives

The objectives of our studies were to determine:

a) the prevalence of chronic toxoplasmosis in population of Moscow city and Moscow region; b) to determine a probable role of the disease in the epidemiology of the RTA in Russian Federation.

Background

Worldwide interest and concern to the emerging problem of toxoplasmosis in general and to its' chronic form in particular had been demonstrated during the last 15-20 years, when new dimensions of the disease had been established. Results of the monitoring of Toxoplasma –infected persons have revealed behavioral changes among them as compared with uninfected persons. Intensity of such changes has closely correlated with the duration of chronic toxoplasmosis. It appears that the mechanism determining the personality changes is associated with an increase in the production of the neurotransmitter dopamine, affecting motor activity, aggression and social behavior of a person. Once in the nerve or inside the muscle tissue, the parasite forms cysts that cause the development of chronic toxoplasmosis. It was found that Toxoplasma localized in the nerve cells of the brain, stimulates the production of dopamine, thus reducing the reaction time and concentration and, thereby, increases the risk of various incidents that have been convincingly demonstrated in epidemiological studies in the Czech Republic and the Republic of Turkey. Subsequent observations among toxoplasmosis - infected persons, conducted in various countries, confirmed the significant behavioral changes,

including personality changes, IQ loss and altered psychomotor activity, particularly increased risk of involvement in road traffic accidents.

Study population and methods of recruitment

In order to establish a general prevalence of chronic Toxoplasmosis among residents of Moscow city, examinations had been carried out among persons attending the Outpatient Department at the Clinical Center of the Sechenov First Moscow State Medical University. No special criteria had been selected for examination in terms of age-, sex- and occupation. Total of 1272 persons had been examined.

The main study represented by two groups: experimental and control.

The experimental group consisted of persons in a possession of valid driving license, and being hospitalized because of a road traffic accident for which they had been held responsible. All persons in the experimental group were the patients of the Sklifosovsky Medical Emergency Institute, Moscow. The criteria for inclusion were:

a) prove that admitted person was driving at the time of accident;

b) evidence that their actions/behavior had led to the occurrence of the accident;

c) age – from 18-45 years.

The criterion for exclusion was driving at the time of an accident under the influence of alcohol/drugs. Total of 100 persons constituted the experimental group – 65 men and 35 women.

The control group consisted of 152 healthy persons aged 18-45 years (82 men and 70 women), who were undertaking routine medical examination at the Clinical Centre of the Sechenov University.

Study design

An analytical epidemiological "case-control" study was carried out. All study patients tested for the presence of IgG and IgM specific antibodies to *Toxoplasma gondii*. Determination of specific immunoglobulin G and M in the blood serum of

the study groups (experimental and control) were determined by enzyme-linked immunosorbent assay (ELISA) test-kits “Vector-Toxo of IgG” and “Vector Toxo-IgM” producer JSC “VECTOR-BEST”. Indicator of chronic toxoplasmosis in patient was a presence of IgG in the absence of IgM. Appendix 1 provides a schematic of the study design.

Statistical analysis

Statistical significance of the results both in the experimental and control groups was obtained through the use of χ^2 criterion, and calculated the odds ratio (OR) with a level of reliability not less than 95%. The Statistical Package EpiInfo Version was employed for calculations.

To assess the strength of the connection between the presence of toxoplasmosis in the experimental and control groups, and the belonging to the compared groups (driver-control), we calculated the Pearson Correlation, which was 0.201 ($p = 0.01$).

Ethics

The study was approve by the Research Ethics Board of Health of the Sechenov First Moscow State Medical University (protocol № 04-13, 10.04.2013). Participants in the experimental and control groups were informed about the purposes of the study, and informed consent in verbal form was obtaine before enrollment in the study.

Results

The prevalence of chronic toxoplasmosis in the residents of Moscow city is presented in table (Appendix 2).

The results of the examination of blood serum in the experimental and control groups are presented in Appendices 2 and 3.

As seen in Table (Appendix 3), the immunoglobulin M was absent in both groups, whereas immunoglobulin G was present in 45% of those tested in the experimental group compared to 26% in the control group. The absence of the immunoglobulin

IgM in conjunction with the presence of IgG suggests the presence of persons with exclusively the chronic form of toxoplasmosis in both the experimental and control groups. It was found that the number of seropositive subjects with IgG in the experimental group was significantly higher than that in the control group. Thus, among the persons involved in traffic accidents who were responsible for their occurrence, the incidence of cases with chronic toxoplasmosis was more than twice that in the control group.

The data in table (Appendix 4) show that the effect of toxoplasmosis was significant and similar for men (OR=2.2, CI95=1.04-4.66, $p<0.02$) and women (OR=2.6, CI95=1.02-6.8,$p<0.02$). There is no statistically significant difference in the ratio of seropositive men ($p<0.02$) and women ($p<0.02$) in the experimental and control groups. It can also be seen that there was practically no difference in the prevalence of chronic toxoplasmosis among men and women in the control group (26.8% and 24.3%, respectively). Almost the same trend was found in the experimental group among men and women, although the level of prevalence was almost 2 times higher compared with the control group.

To assess the strength of the connection between the presence of toxoplasmosis in the experimental and control groups, and the belonging to the compared groups (driver-control), we calculated the Pearson Correlation, which was 0.201 ($p = 0.01$).

The data in table (Appendix 5) show that there was no statistically significant difference between the studied groups with respect to sex and age.

The partial correlation coefficient with respect to age and sex was calculated as 0.202 ($p=0.01$). Similar results were obtained in a stratified analysis using an estimate for the overall odds ratio as the Mantel-Haenszel Common Odds Ratio Estimate, when stratified by sex. The adjusted OR was 2.35 CI 1.37-4.03.

Appendix 1. The block diagram of the study design.

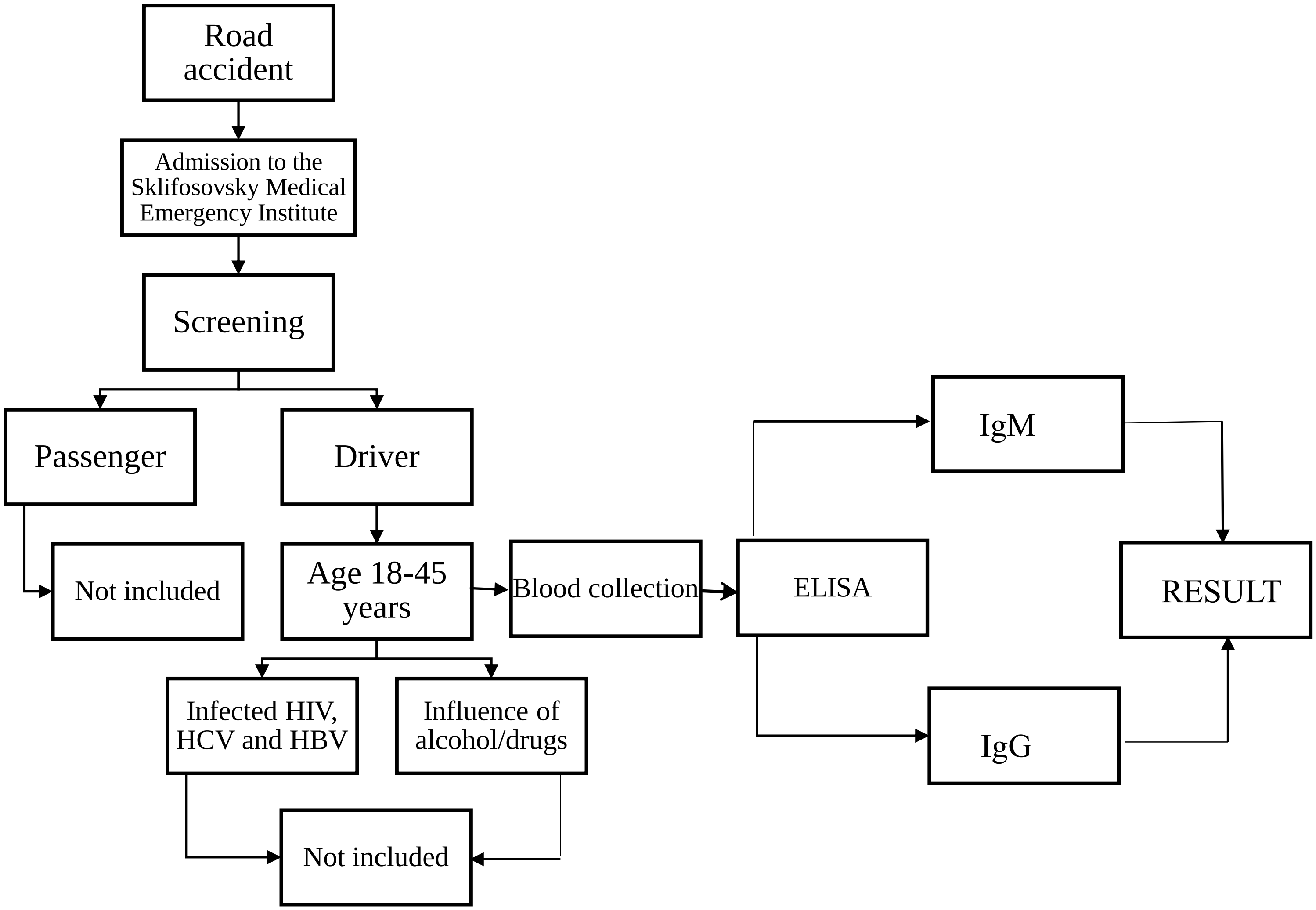

Appendix 2. Prevalence of Chronic Toxoplasmosis Among the Residents of Moscow City (Russian Federation), 2015.

Group	Examined	Positive results (ELISA test)	
		Absolute number	Percent (%)
Total number	1272	323	25.39
Men	497	120	24.14
Women	775	203	26.19

Appendix 3. Comparative Prevalence of Immunoglobulins M and G to *Toxoplasma gondii* in the Experimental Group and the Control Group.

Group	examined	Absolute number		Positive results (%)	Odds ratio	C.I.$_{95}$	p-values
		IgM	IgG				
Case	100	0	45	45.00	2.37	1.34-4.2	0.001
Control	152	0	39	25.66			

Appendix 4.
Comparative Gender Prevalence of Chronic Toxoplasmosis in the Experimental Group and the Control Group.

Group	Experimental group			Control group			Odds ratio	C.I.$_{95}$	p-values
	Examined	Positive results		Examined	Positive results				
		Absolute number	Percent (%)		Absolute number	Percent (%)			
Men	65	29	44.61	82	22	26.83	2.2	1.04-4.66	0.02
Women	35	16	45.71	70	17	24.28	2.63	1.02-6.8	0.02

Appendix 5. The Results of Multivariate Analysis.

Factor	Total (252)	Experimental (100)	Control (152)	p-values
Men	147<br>58,3%	65<br>65,0%	82<br>53,9%	p=0,091
Women	105<br>41,7%	35<br>35,0%	70<br>46,1%	
Age	34,30<br>SE=7,11<br>SD=0,45	34,31<br>SE=7,77<br>SD=0,78	34,50<br>SE=6,66<br>SD=0,54	p=0,836

Appendix 6. Experimental Group

N of sample	sex	results	age
104892	famale	negative	43
104878	male	positive	35
105129	famale	negative	22
106612	male	positive	35
106613	male	negative	45
108133	male	negative	21
108378	male	negative	31
108380	male	negative	28
108512	male	negative	42
109014	male	negative	26
109034	male	positive	41
108942	male	positive	31
108943	male	positive	25
111024	male	negative	20
111001	male	negative	28
111191	male	negative	21
111174	male	negative	40
111318	male	positive	39
111294	male	negative	39
111491	male	negative	33
109147	male	positive	31
109316	male	positive	25
109312	male	positive	42
110410	male	positive	44
110451	male	negative	24
111679	male	negative	43
111681	male	positive	43
111882	famale	negative	23
111851	male	negative	39

112137	male	positive	41
112284	male	positive	31
112446	male	negative	45
112425	male	positive	45
112410	male	negative	31
112695	male	positive	44
112696	male	negative	30
113058	male	positive	36
113250	male	negative	33
113219	male	positive	31
113501	male	negative	29
113474	male	positive	29
113519	male	negative	27
114311	male	positive	32
114356	male	negative	43
114409	male	positive	19
144512	male	negative	23
114892	male	positive	34
114924	male	positive	23
115089	male	negative	39
115020	male	positive	24
115419	male	negative	45
115899	male	negative	44
115779	male	negative	42
116070	male	negative	42
116069	male	negative	33
3116369	male	negative	26
3116373	male	positive	34
116490	male	negative	31
116669	male	positive	33
116695	male	negative	29
116707	famale	positive	18
116982	male	negative	37
116984	male	positive	26
117177	male	negative	37
117315	famale	positive	44
118099	male	positive	33
118216	male	negative	29
123399	famale	positive	27
127043	male	positive	26
127046	male	negative	30
128969	famale	positive	40
128885	male	positive	38
104485	famale	positive	41
104877	famale	negative	42
105914	famale	negative	21

105956	famale	negative	39
106667	famale	negative	40
108222	famale	negative	29
108359	famale	positive	44
108360	famale	positive	41
108361	famale	negative	24
109021	famale	negative	30
111007	famale	negative	43
111050	famale	negative	42
111508	famale	negative	37
109162	famale	positive	45
111680	famale	negative	44
112522	famale	positive	45
112637	famale	negative	35
113517	famale	positive	42
114652	famale	negative	38
114307	famale	positive	27
114621	famale	negative	28
114789	famale	positive	45
115019	famale	positive	24
115087	famale	positive	38
115861	famale	negative	41
116233	famale	positive	35
3116382	famale	negative	45
3116378	famale	positive	34

Appendix 7. Control Group

N of sample	sex	results	age
1	male	positive	40
2	male	positive	41
3	male	negative	34
4	male	negative	38
5	male	negative	34
6	male	negative	26
7	male	negative	30
8	male	negative	34
9	male	negative	41
10	male	negative	34
11	male	negative	34
12	male	negative	38
13	male	positive	34
14	male	positive	41
15	male	positive	45
16	male	positive	28

17	male	negative	45
18	male	negative	45
19	male	negative	32
20	male	negative	45
21	male	negative	45
22	male	negative	45
23	male	negative	42
24	male	negative	36
25	male	negative	41
26	male	negative	42
27	male	negative	42
28	male	negative	42
29	male	negative	38
30	male	negative	39
31	male	negative	34
32	male	positive	39
33	male	negative	32
34	male	negative	39
35	male	negative	39
36	male	negative	39
37	male	positive	27
38	male	positive	31
39	male	positive	43
40	male	positive	41
41	male	positive	41
42	male	negative	43
43	male	negative	43
44	male	negative	41
45	male	negative	43
46	male	negative	20
47	male	negative	41
48	male	negative	37
49	male	negative	43
50	male	negative	32
51	male	negative	29
52	male	negative	31
53	male	negative	26
54	male	negative	43
55	male	negative	43
56	male	negative	26
57	male	negative	43
58	male	positive	43
59	male	positive	29
60	male	positive	43
61	male	positive	43
62	male	positive	30

63	male	negative	30
64	male	negative	30
65	male	positive	26
66	male	positive	26
67	male	positive	30
68	male	negative	30
69	male	negative	28
70	male	negative	37
71	male	negative	31
72	male	negative	30
73	male	negative	41
74	male	negative	30
75	male	negative	27
76	male	positive	30
77	male	positive	30
78	male	negative	21
79	male	negative	39
80	male	negative	30
81	male	negative	41
82	male	negative	30
83	female	positive	30
84	female	negative	30
85	female	positive	28
86	female	negative	37
87	female	negative	31
88	female	negative	30
89	female	negative	41
90	female	negative	30
91	female	negative	27
92	female	negative	30
93	female	positive	30
94	female	negative	21
95	female	negative	39
96	female	negative	30
97	female	negative	41
98	female	positive	30
99	female	positive	41
100	female	negative	43
101	female	negative	20
102	female	negative	41
103	female	negative	37
104	female	negative	43
105	female	negative	32
106	female	positive	29
107	female	negative	31
108	female	negative	26

109	female	negative	43
110	female	positive	43
111	female	negative	26
112	female	positive	43
113	female	negative	43
114	female	negative	29
115	female	negative	43
116	female	positive	43
117	female	negative	30
118	female	negative	42
119	female	positive	36
120	female	negative	41
121	female	negative	42
122	female	negative	42
123	female	positive	42
124	female	negative	38
125	female	negative	39
126	female	positive	34
127	female	negative	39
128	female	negative	32
129	female	negative	41
130	female	negative	43
131	female	negative	20
132	female	positive	41
133	female	negative	37
134	female	negative	43
135	female	positive	32
136	female	negative	29
137	female	negative	31
138	female	negative	26
139	female	negative	43
140	female	negative	43
141	female	negative	26
142	female	positive	43
143	female	negative	43
144	female	negative	29
145	female	negative	36
146	female	positive	31
147	female	negative	29
148	female	negative	37
149	female	negative	34
150	female	negative	22
151	female	positive	41
152	female	negative	33

Appendix 8. Prevalence of Chronic Toxoplasmosis Among the Residents of Moscow City 112 (Russian Federation), 2015

N of sample	sex	results	age
65595	male	positive	20
65598	female	positive	39
65603	female	positive	55
65604	female	positive	20
65610	male	positive	41
65614	female	positive	20
65615	female	positive	37
65616	male	negative	29
65622	female	negative	31
65628	female	positive	20
65633	female	positive	33
65639	female	positive	33
65642	female	positive	20
65645	female	negative	69
65646	female	negative	63
65647	female	negative	47
65652	female	positive	58
65653	female	negative	20
65655	female	negative	40
65657	male	positive	35
65661	male	negative	62
65662	female	positive	45
65664	female	negative	29
65666	male	negative	20
65667	female	positive	20
65668	female	positive	34
65673	male	negative	36
65674	female	negative	55
65675	male	positive	39
65682	female	positive	22
65686	female	negative	31
65687	male	negative	27
65695	female	negative	66
65698	female	negative	33
65699	female	negative	20
65708	male	negative	20
65710	female	negative	43
65711	female	positive	31
65713	female	negative	50
65717	male	negative	20
65721	female	negative	31
65728	female	negative	45

65729	female	negative	20
65730	female	negative	67
65738	female	negative	20
65741	female	negative	30
65743	female	negative	44
65749	female	negative	25
65752	male	negative	20
65754	female	negative	20
65755	male	positive	48
65756	female	positive	18
65758	male	negative	20
65759	female	negative	39
65763	female	negative	31
65764	male	negative	20
65765	female	negative	20
65766	female	negative	44
65768	female	negative	45
65770	female	negative	46
65772	female	positive	46
65774	female	positive	20
65779	female	positive	36
65790	female	negative	48
65791	male	negative	29
65793	male	negative	34
65796	female	negative	24
65797	male	negative	39
65804	male	negative	19
65807	male	negative	20
65809	female	negative	36
65811	female	negative	18
65812	female	positive	20
65816	male	negative	20
65818	female	negative	50
65822	female	negative	20
65823	female	negative	40
65827	female	negative	36
65828	male	negative	39
65829	male	negative	20
65830	male	negative	20
65832	female	negative	32
65833	male	negative	22
65835	male	negative	20
65839	male	positive	35
65840	male	negative	35
65842	male	negative	33
65843	female	negative	37

65844	male	negative	24
65846	male	negative	18
65847	male	negative	54
65848	female	positive	24
65853	female	negative	34
65854	female	negative	66
65857	male	negative	35
65858	male	negative	27
65860	female	positive	35
65861	female	negative	28
65869	female	positive	61
65871	female	positive	36
65872	male	positive	29
65875	male	negative	46
65876	male	positive	26
65877	female	positive	35
65878	female	positive	27
65879	female	negative	32
65882	male	negative	45
65885	female	negative	35
65891	female	negative	31
65892	female	negative	57
65898	female	negative	63
65899	male	negative	35
65906	female	negative	46
65910	male	negative	18
65911	female	negative	70
65916	male	positive	35
65918	male	positive	34
65930	female	positive	36
65931	male	positive	35
65933	male	negative	40
65936	male	negative	35
65939	male	negative	30
65942	male	negative	35
65950	female	negative	72
65954	male	negative	35
65956	female	positive	35
65959	female	positive	35
65960	female	negative	23
65961	female	negative	41
65962	female	negative	35
65969	female	negative	35
65970	female	negative	35
65971	female	negative	29
65973	female	positive	64

65977	female	positive	35
65979	male	negative	22
65982	male	negative	35
65986	male	negative	40
65990	female	negative	35
65991	female	negative	49
65993	female	negative	70
65994	female	negative	49
65996	female	positive	53
65997	female	negative	37
65998	male	negative	35
66004	female	negative	55
66007	male	negative	35
66008	female	negative	22
66010	female	negative	35
66012	male	positive	35
66017	female	negative	29
66021	female	negative	60
66023	male	negative	31
66026	female	negative	35
66028	male	negative	35
66031	female	positive	52
66035	female	negative	36
66038	male	negative	61
66039	female	negative	29
66040	female	positive	35
66042	female	positive	35
66046	female	negative	26
66047	female	negative	56
66050	male	positive	37
66054	female	negative	34
66059	female	negative	37
66065	female	negative	37
66066	female	positive	26
66067	female	negative	54
66071	female	negative	28
66074	male	negative	32
66075	female	positive	30
66080	male	negative	52
66088	female	negative	35
66101	male	positive	27
66104	female	negative	37
66105	male	positive	39
66107	female	negative	28
66108	female	negative	51
66109	female	negative	29

66119	female	positive	36
66126	male	negative	34
66128	female	negative	44
66130	female	negative	45
66135	female	negative	46
66139	female	negative	54
66140	female	negative	42
66142	female	negative	31
66144	female	negative	29
66146	male	positive	35
66149	female	negative	35
66151	female	negative	31
66155	male	negative	25
66158	female	negative	27
66161	female	positive	47
66167	female	negative	41
66168	female	negative	27
66172	female	negative	28
66173	female	negative	29
66175	male	negative	41
66176	female	negative	52
66179	female	positive	33
66180	male	negative	35
66181	male	negative	35
66182	male	negative	65
66183	female	negative	35
66186	female	negative	35
66188	female	negative	27
66189	female	negative	79
66193	female	negative	35
66195	female	negative	36
66196	female	positive	31
66197	male	negative	35
66198	female	negative	62
66199	female	negative	29
66200	male	positive	57
66202	female	negative	24
66204	female	negative	38
66205	female	negative	35
66207	male	positive	33
66209	male	negative	61
66213	female	positive	29
66218	female	positive	41
66223	female	negative	33
66226	female	negative	30
66227	male	negative	35

66228	male	negative	35
66230	male	negative	35
66233	female	negative	35
66234	female	negative	31
66236	male	negative	35
66237	female	negative	35
66238	male	negative	35
66239	female	negative	38
66242	female	negative	26
66243	female	negative	35
66244	female	negative	32
66245	female	positive	54
66252	male	negative	35
66253	male	negative	32
66255	male	negative	35
66257	female	positive	35
66260	female	negative	35
66262	female	negative	28
66263	female	negative	74
66264	female	negative	35
66269	male	negative	38
66270	male	negative	35
66272	female	negative	38
66275	female	negative	57
66279	female	negative	35
66281	female	negative	35
66286	female	negative	32
66298	female	positive	34
66302	male	negative	56
66303	male	negative	43
66304	female	negative	35
66306	female	negative	46
66309	female	negative	51
66310	female	negative	35
66315	male	positive	21
66317	female	negative	61
66318	female	negative	39
66319	female	negative	44
66322	female	negative	24
66328	male	negative	25
66331	female	negative	35
66332	female	positive	23
66333	male	negative	35
66334	female	negative	41
66335	female	negative	41
66338	male	negative	35

66342	female	negative	40
66343	female	positive	58
66350	female	positive	41
66351	female	positive	18
66357	female	negative	35
66359	female	negative	35
66363	male	negative	28
66370	female	negative	37
66374	female	positive	35
66375	female	negative	30
66376	female	negative	35
66377	female	negative	32
66378	male	positive	31
66380	male	negative	35
66381	male	negative	35
66383	male	negative	35
66388	male	positive	35
66390	female	negative	44
66396	female	negative	35
66397	female	negative	35
66399	female	negative	33
66405	male	negative	35
66411	male	positive	56
66413	male	negative	34
66414	male	positive	50
66415	male	positive	51
66416	male	negative	40
66417	male	negative	35
66419	female	positive	35
66425	male	negative	40
66429	female	negative	35
66431	female	positive	35
66436	male	positive	23
66437	male	positive	28
66442	female	negative	37
66443	male	negative	35
66444	male	negative	35
66445	male	negative	35
66447	male	negative	57
66448	female	positive	28
66455	female	negative	51
66456	female	negative	31
66460	female	negative	35
66462	female	negative	35
66463	female	positive	31
66465	male	negative	35

66473	female	negative	35
66482	female	positive	57
66487	female	negative	32
66489	male	negative	35
66491	female	positive	21
66495	female	negative	35
66497	male	positive	35
66500	female	negative	35
66507	female	negative	43
66508	female	positive	30
66510	male	negative	29
66513	female	negative	31
66515	female	positive	42
66517	male	negative	59
66518	male	negative	35
66519	female	positive	35
66529	female	negative	31
66530	male	negative	24
66531	female	negative	20
66535	female	positive	57
66537	male	negative	26
66545	male	negative	58
66547	male	negative	37
66550	female	negative	19
66553	male	negative	24
66554	male	negative	25
66555	female	negative	46
66557	female	negative	35
66558	female	negative	52
66559	female	negative	24
66565	female	negative	41
66566	female	negative	32
66568	female	negative	35
66570	male	negative	35
66571	female	negative	28
66573	female	positive	30
66575	male	negative	35
66576	female	negative	31
66579	female	negative	69
66580	female	positive	47
66581	female	negative	53
66582	female	negative	35
66583	female	positive	44
66584	female	negative	53
66585	male	negative	25
66586	female	negative	43

66587	female	negative	29
66588	female	negative	27
66589	male	negative	32
66591	male	positive	29
66592	female	positive	46
66593	male	negative	48
66598	female	positive	62
66600	male	negative	35
66602	male	negative	35
66607	male	negative	18
66611	female	negative	41
66613	female	positive	45
66616	female	negative	23
66617	female	negative	49
66620	female	negative	67
66623	female	positive	25
66629	female	negative	24
66631	male	negative	35
66632	male	negative	35
66633	male	negative	36
66641	female	negative	31
66648	female	negative	38
66649	female	negative	58
66650	female	negative	35
66654	male	negative	26
66656	female	negative	32
66657	female	positive	35
66659	female	negative	34
66660	male	negative	35
66664	female	positive	41
66665	female	negative	25
66670	male	negative	23
66671	female	negative	42
66674	male	negative	35
66681	female	negative	35
66688	male	negative	34
66697	male	negative	53
66703	female	negative	22
66704	female	negative	29
66714	male	negative	54
66723	male	negative	25
66726	male	positive	29
66728	male	negative	43
66730	female	positive	29
66731	male	negative	29
66742	female	negative	24

66751	female	positive	34
66752	female	negative	29
66754	male	negative	29
66757	female	negative	32
66760	male	negative	29
66763	male	negative	29
66771	male	negative	36
66772	female	negative	48
66774	male	negative	29
66780	female	negative	47
66781	male	negative	33
66783	female	negative	29
66784	male	negative	43
66787	male	negative	22
66789	male	negative	22
66790	female	negative	52
66791	male	negative	22
66793	female	negative	28
66794	female	positive	22
66796	male	negative	25
66799	female	negative	68
66801	female	negative	60
66802	female	positive	56
66805	female	positive	61
66809	male	positive	49
66816	male	negative	26
66820	female	positive	22
66821	female	negative	36
66822	female	negative	43
66824	female	negative	34
66825	male	negative	22
66829	male	positive	44
66830	male	positive	26
66831	female	positive	47
66839	female	negative	40
66842	female	negative	22
66847	female	negative	57
66851	male	positive	45
66853	female	positive	22
66857	female	negative	28
66860	male	positive	22
66862	female	positive	20
66865	female	positive	32
66869	female	positive	22
66875	female	positive	53
66876	female	positive	22

66879	male	positive	22
66880	female	positive	22
66883	male	positive	48
66885	female	positive	31
66886	male	positive	22
66888	male	positive	34
66889	male	positive	22
66890	female	negative	43
66894	male	negative	31
66897	male	negative	42
66899	female	negative	68
66904	female	negative	19
66909	female	negative	22
66910	male	negative	32
66918	female	negative	36
66921	female	negative	35
66925	male	negative	22
66927	female	negative	22
66929	female	negative	30
66930	female	negative	30
66931	female	negative	28
66934	female	negative	22
66941	female	negative	38
66942	female	negative	39
66943	male	negative	24
66946	female	negative	41
66947	female	negative	55
66952	female	negative	22
66953	female	negative	32
66954	female	negative	75
66962	male	negative	22
66966	female	negative	28
66969	female	negative	22
66970	female	negative	60
66971	male	negative	24
66978	male	negative	40
66980	female	negative	19
66981	female	negative	35
66982	male	negative	31
66988	male	negative	57
66991	female	negative	22
67002	female	positive	27
67004	male	negative	45
67008	female	positive	71
67009	male	negative	22
67011	female	negative	61

67016	female	negative	33
67025	female	positive	51
67031	female	negative	27
67038	female	positive	33
67039	male	negative	22
67043	male	positive	46
67044	female	negative	52
67046	male	negative	30
67048	female	negative	42
67049	female	negative	22
67051	male	negative	22
67055	male	negative	25
67056	male	negative	45
67057	male	negative	18
67059	female	negative	53
67060	female	positive	35
67068	female	negative	34
67072	female	positive	28
67077	male	negative	22
67078	male	negative	22
67080	female	negative	22
67093	female	negative	22
67094	female	negative	31
67097	female	negative	42
67098	male	negative	45
67100	female	negative	48
67101	female	negative	27
67103	female	negative	40
67105	female	positive	33
67107	female	negative	22
67110	male	positive	44
67111	male	negative	22
67117	male	negative	22
67121	female	positive	36
67123	female	positive	28
67128	female	positive	61
67130	female	positive	43
67132	male	negative	22
67134	female	negative	58
67139	female	negative	20
67140	female	negative	48
67147	female	positive	31
67148	female	negative	30
67149	female	negative	30
67152	female	negative	29
67154	female	negative	30

67156	female	positive	22
67162	male	negative	35
67163	female	positive	31
67174	male	positive	22
67176	female	negative	22
67177	female	negative	47
67183	female	positive	70
67184	female	negative	28
67185	female	negative	22
67187	female	negative	22
67189	female	negative	27
67196	female	positive	53
67201	female	negative	22
67206	female	negative	22
67207	female	positive	47
67208	male	negative	22
67210	female	positive	18
67211	female	positive	55
67216	male	negative	22
67222	female	negative	22
67227	male	negative	22
67229	female	negative	31
67230	female	negative	34
67237	female	negative	34
67238	female	negative	76
67239	male	negative	22
67243	female	negative	41
67261	female	negative	28
67262	female	negative	30
67266	female	negative	40
67267	male	negative	20
67271	female	negative	42
67272	male	negative	49
67273	female	positive	40
67279	female	negative	40
67280	male	positive	40
67281	female	negative	18
67282	female	negative	40
67286	female	negative	40
67291	female	negative	37
67293	male	negative	42
67298	female	negative	40
67299	female	negative	25
67301	female	positive	36
67302	female	negative	40
67304	female	positive	70

67305	female	negative	49
67312	female	positive	55
67313	female	positive	53
67314	female	negative	31
67316	female	positive	29
67319	female	negative	56
67320	female	negative	30
67327	female	negative	45
67328	male	negative	40
67330	female	negative	40
67332	female	negative	31
67337	male	negative	40
67338	female	negative	37
67348	female	positive	25
67350	male	negative	40
67353	male	negative	54
67354	female	positive	34
67355	female	negative	21
67357	male	negative	40
67359	male	negative	40
67366	female	negative	32
67367	male	negative	37
67370	female	negative	19
67387	male	negative	35
67391	female	negative	40
67393	female	negative	40
67395	male	positive	34
67398	female	positive	61
67399	male	negative	45
67400	male	negative	31
67402	female	negative	64
67407	female	negative	20
67408	male	negative	19
67409	female	negative	56
67410	female	positive	32
67413	female	negative	21
67417	female	negative	46
67420	female	negative	40
67421	female	negative	40
67422	female	negative	34
67423	female	negative	40
67428	male	positive	40
67429	female	negative	44
67437	female	negative	34
67442	male	negative	34
67443	female	positive	28

67444	male	negative	34
67445	male	negative	40
67446	male	positive	40
67449	male	negative	40
67451	female	positive	38
67454	female	positive	45
67455	male	positive	19
67458	female	negative	34
67461	male	positive	49
67472	female	positive	50
67475	male	negative	40
67477	female	negative	58
67478	female	negative	36
67479	female	negative	28
67480	male	negative	40
67484	female	negative	40
67485	female	positive	68
67486	male	negative	27
67488	female	negative	31
67491	male	negative	40
67493	female	negative	47
67494	female	negative	40
67495	female	negative	69
67496	female	positive	44
67497	male	negative	40
67498	male	negative	40
67499	female	positive	35
67502	female	negative	40
67507	female	negative	33
67508	female	negative	34
67509	male	negative	33
67510	female	negative	32
67511	female	negative	32
67522	male	negative	67
67524	female	positive	39
67528	female	negative	29
67529	male	negative	44
67530	male	negative	40
67533	female	negative	49
67535	female	positive	40
67536	female	positive	44
67541	female	negative	40
67555	female	negative	40
67557	female	negative	31
67559	male	negative	36
67561	female	negative	50

67563	female	negative	38
67568	female	negative	40
67579	female	negative	40
67584	female	negative	40
67591	female	positive	25
67596	male	negative	36
67606	male	negative	40
67607	female	negative	40
67608	female	negative	29
67609	male	negative	18
67610	male	positive	40
67614	male	negative	40
67616	female	negative	19
67618	female	negative	29
67620	male	negative	40
67621	female	negative	34
67634	female	positive	47
67637	female	positive	33
67640	male	negative	40
67645	female	positive	51
67648	female	positive	39
67650	male	positive	64
67651	female	negative	35
67658	female	negative	40
67660	female	negative	59
67662	female	negative	40
67663	female	positive	44
67665	male	negative	40
67666	male	negative	32
67678	female	positive	38
67679	female	negative	33
67682	female	positive	38
67683	female	positive	20
67690	male	negative	42
67694	female	negative	32
67696	female	negative	42
67702	female	negative	37
67703	female	negative	32
67706	female	positive	56
67707	male	negative	32
67709	female	negative	32
67713	female	negative	26
67718	female	negative	34
67720	female	positive	26
67722	male	negative	32
67725	female	negative	18

67728	male	negative	32
67729	male	negative	36
67733	female	positive	32
67744	male	negative	49
67746	male	negative	32
67747	male	negative	32
67750	male	negative	32
67751	male	positive	47
67753	female	negative	57
67755	female	negative	32
67758	male	negative	32
67763	male	negative	44
67764	male	negative	33
67768	female	negative	32
67779	male	negative	44
67780	male	negative	31
67790	female	negative	32
67794	male	negative	32
67799	male	positive	60
67804	male	negative	32
67811	male	negative	32
67829	female	positive	67
67833	female	negative	32
67835	female	negative	50
67836	female	positive	68
67840	female	negative	32
67842	female	negative	32
67843	male	negative	27
67850	male	negative	43
67851	female	negative	32
67852	male	negative	28
67858	female	negative	32
67867	male	negative	32
67869	male	positive	56
67874	female	negative	32
67875	female	positive	73
67883	male	negative	41
67890	male	negative	32
67891	female	negative	38
67894	female	negative	49
67902	female	negative	31
67904	female	negative	38
67906	female	negative	26
67914	female	positive	47
67921	male	negative	22
67922	female	negative	39

67924	female	negative	32
67925	male	negative	23
67932	female	negative	31
67939	female	negative	21
67940	female	negative	32
67941	male	negative	32
67942	female	negative	32
67947	female	positive	23
67955	male	positive	53
67967	female	negative	26
67968	male	positive	40
67969	female	positive	29
67973	female	positive	42
67977	female	negative	32
67985	female	negative	21
67986	male	positive	32
67989	female	positive	60
67999	female	negative	32
68000	male	negative	32
68004	female	negative	48
68012	female	negative	32
68014	female	negative	19
68018	female	negative	39
68019	male	negative	32
68020	male	negative	32
68025	female	negative	33
68026	female	positive	42
68027	female	negative	32
68033	female	negative	24
68037	male	negative	32
68044	male	positive	50
68053	male	negative	35
68054	male	negative	48
68056	male	negative	26
68057	female	negative	32
68058	female	positive	48
68066	female	negative	31
68080	male	positive	42
68083	female	negative	29
68087	female	negative	39
68090	female	negative	32
68091	male	negative	19
68092	male	negative	32
68093	male	negative	32
68094	female	negative	34
68096	female	negative	32

68103	female	positive	62
68108	female	negative	65
68118	male	positive	35
68126	female	positive	50
68129	female	negative	32
68130	female	negative	55
68135	male	negative	32
68140	male	negative	32
68149	male	negative	32
68150	female	positive	57
68151	female	negative	32
68152	male	negative	32
68159	female	negative	32
68164	female	positive	32
68167	female	negative	32
68173	female	negative	32
68174	male	negative	32
68181	female	negative	31
68184	female	positive	41
68197	female	negative	22
68213	female	positive	53
68215	female	negative	32
68216	male	positive	37
68225	male	negative	34
68228	male	positive	44
68231	male	positive	32
68236	male	negative	39
68240	female	positive	56
68245	female	negative	39
68247	female	negative	32
68253	female	negative	36
68255	male	negative	32
68256	female	negative	41
68260	female	negative	28
68269	male	positive	32
68271	female	positive	65
68273	female	negative	32
68277	female	positive	25
68279	female	negative	32
68282	male	negative	31
68287	female	negative	32
68288	female	positive	28
68292	female	positive	35
68297	female	negative	32
68299	female	negative	49
68301	male	negative	32

68310	female	negative	40
68311	male	positive	35
68313	male	positive	32
68314	male	positive	24
68321	female	negative	42
68331	female	negative	32
68336	male	negative	36
68337	male	negative	32
68340	male	negative	32
68342	male	negative	32
68344	female	negative	35
68347	female	negative	59
68350	female	negative	32
68351	female	negative	32
68355	female	negative	52
68361	male	negative	38
68363	male	positive	38
68367	female	negative	32
68373	female	positive	62
68374	female	positive	56
68377	male	negative	32
68378	female	positive	32
68380	male	negative	27
68381	male	negative	32
68386	female	positive	32
68389	female	negative	32
68391	female	negative	32
68392	male	negative	32
68396	female	negative	32
68398	male	positive	29
68399	female	negative	34
68405	female	positive	37
68410	female	positive	40
68414	male	negative	43
68419	male	negative	42
68420	female	negative	42
68432	male	positive	35
68434	male	negative	42
68435	male	negative	42
68445	male	positive	28
68452	female	positive	25
68456	female	negative	42
68458	male	negative	42
68466	male	positive	29
68468	male	negative	40
68474	male	negative	42

68477	male	positive	33
68488	male	negative	42
68494	female	negative	42
68499	female	negative	42
68502	male	negative	36
68508	female	negative	36
68510	female	negative	42
68512	female	negative	33
68513	female	negative	42
68514	female	negative	42
68515	female	negative	34
68516	female	negative	34
68517	female	negative	42
68518	female	negative	42
68522	female	negative	42
68523	female	negative	51
68525	male	positive	42
68526	female	negative	39
68528	female	negative	51
68529	male	negative	28
68531	female	negative	44
68538	female	positive	53
68545	female	negative	24
68546	male	negative	42
68548	female	negative	55
68549	female	negative	49
68551	male	positive	41
68552	female	negative	26
68553	male	negative	22
68554	male	negative	42
68556	female	negative	30
68562	female	negative	42
68564	male	negative	42
68570	male	positive	42
68571	female	positive	45
68573	male	negative	42
68574	female	positive	48
68588	male	negative	42
68590	female	negative	30
68592	male	negative	42
68595	female	negative	29
68596	female	negative	45
68599	male	negative	42
68601	male	negative	42
68604	female	positive	51
68610	male	negative	42

68611	male	negative	42
68613	male	negative	53
68615	female	positive	37
68618	female	negative	42
68630	female	negative	36
68631	female	negative	42
68639	female	negative	27
68640	male	negative	45
68643	female	negative	62
68644	female	negative	42
68649	male	negative	42
68656	female	negative	42
68664	female	negative	40
68667	male	negative	34
68670	male	negative	42
68671	female	positive	56
68672	female	negative	42
68675	male	negative	42
68678	female	negative	32
68680	male	negative	42
68681	female	positive	66
68683	male	negative	42
68685	male	positive	39
68687	female	negative	42
68693	female	negative	42
68694	female	negative	42
68708	male	negative	40
68714	female	negative	42
68715	male	negative	29
68721	female	negative	42
68722	male	negative	42
68723	female	negative	42
68731	female	negative	22
68732	male	positive	63
68736	male	negative	42
68738	male	negative	42
68740	male	positive	36
68744	female	positive	66
68752	female	negative	42
68753	female	positive	27
68754	female	negative	42
68759	female	positive	32
68760	female	negative	42
68761	female	negative	32
68762	male	negative	42
68764	male	negative	42

68765	male	negative	42
68767	female	positive	31
68768	female	negative	39
68780	male	negative	34
68790	male	negative	35
68848	male	negative	42
68850	male	positive	33
68855	male	positive	52
68922	female	negative	21
68928	female	negative	24
68931	male	negative	42
68941	male	positive	42
68994	female	positive	36
69027	female	negative	50
69039	female	negative	18
69046	male	negative	19
69083	female	positive	41
69086	female	negative	42
69094	male	negative	42
69112	male	negative	60
69147	female	negative	19
69174	male	negative	42
69175	female	negative	42
69176	female	negative	28
69182	female	negative	42
69185	female	negative	42
69197	female	positive	29
69210	female	positive	60
69211	female	negative	42
69215	male	positive	32
69216	female	positive	35
69220	female	negative	42
69223	male	negative	20
69224	female	negative	49
69228	female	negative	33
69234	female	negative	42
69235	female	negative	30
69236	female	negative	42
69243	female	negative	33
69245	female	negative	19
69248	female	negative	25
69252	male	negative	36
69254	female	negative	42
69256	male	negative	42
69258	male	positive	58
69259	female	negative	27

69263	male	negative	42
69267	male	negative	42
69269	female	negative	38
69271	female	positive	64
69272	female	positive	38
69275	male	negative	42
69282	male	negative	23
69286	female	negative	56
69287	female	negative	42
69288	female	negative	18
69290	male	negative	42
69291	female	negative	42
69293	male	negative	37
69296	male	negative	42
69302	female	positive	58
69305	female	negative	42
69307	female	negative	42
69308	female	negative	42
69316	female	positive	33
69326	female	positive	58
69328	female	negative	18
69335	male	negative	30
69346	female	positive	41
69350	male	negative	42
69355	female	negative	31
69357	female	positive	58
69371	female	negative	42
69372	male	negative	26
69373	female	negative	52
69376	female	positive	30
69377	female	negative	42
69378	female	positive	42
69380	female	negative	42
69384	male	positive	45
69385	female	positive	46
69388	female	positive	58
69392	male	negative	21
69397	male	negative	42
69406	female	negative	24
69412	female	negative	24
69413	female	negative	24
69414	female	positive	53
69415	male	positive	64
69421	female	negative	24
69422	female	negative	51
69424	female	negative	36

69425	female	negative	24
69430	male	negative	22
69435	male	negative	55
69436	female	negative	43
69437	female	negative	55
69450	female	negative	37
69460	female	negative	19
69462	female	positive	51
69463	female	positive	56
69469	female	positive	35
69470	female	positive	40
69471	female	positive	30
69472	female	negative	24
69474	female	negative	31
69477	female	negative	59
69483	male	negative	24
69486	female	negative	71
69493	male	negative	24
69499	male	negative	30
69501	female	negative	24
69507	male	negative	24
69508	female	negative	24
69510	male	negative	24
69512	female	negative	24
69513	female	negative	53
69519	female	negative	31
69524	female	negative	24
69531	female	negative	25
69533	female	negative	24
69534	female	negative	56
69535	female	negative	24
69537	male	negative	22
69540	female	negative	24
69541	female	negative	53
69543	female	negative	51
69544	male	negative	26
69549	female	negative	66
69550	female	positive	53
69561	male	negative	33
69562	female	negative	43
69563	female	negative	63
69564	male	negative	47
69566	male	positive	24
69568	female	negative	24
69574	female	negative	24
69579	female	negative	24

69580	female	negative	24
69586	female	positive	47
69587	female	negative	24
69588	male	negative	24
69596	female	negative	24
69600	male	positive	53
69601	male	negative	29
69602	male	negative	29
69605	female	negative	32
69608	female	negative	29
69610	female	positive	34
69611	female	negative	35
69614	male	positive	77
69624	female	negative	25
69626	male	negative	29
69631	male	negative	29
69633	male	negative	29
69637	male	negative	29
69640	female	negative	29
69644	female	negative	32
69646	male	negative	22
69649	female	negative	29
69652	male	negative	29
69654	female	negative	45
69656	female	negative	28
69657	female	negative	29
69662	male	negative	25
69669	female	negative	29
69671	male	negative	51
69672	female	negative	52
69674	male	negative	35
69675	male	negative	29
69679	male	negative	42
69683	female	negative	29
69687	male	negative	29
69689	female	negative	29
69690	female	negative	37
69693	male	negative	25
69697	female	negative	29
69698	female	negative	29
69699	male	negative	29
69701	female	negative	58
69704	female	positive	26
69713	male	negative	44
69717	male	negative	29
69721	male	negative	21

69722	female	negative	45
69723	male	positive	54
69725	male	negative	29
69726	male	negative	29
69727	male	negative	36
69728	female	negative	40
69730	female	negative	44
69740	female	positive	29
69745	female	negative	20
69746	female	positive	48
69747	male	positive	29
69751	female	negative	29
69752	female	negative	29
69753	female	negative	29
69754	female	negative	32
69756	female	negative	29
69779	male	positive	28
69788	male	positive	49
69819	male	negative	40
69827	male	positive	33
69851	male	negative	40
69869	male	negative	40
69885	male	negative	40
69887	male	negative	33
69897	male	positive	36
69901	male	positive	40
69909	male	positive	24
69928	male	negative	40
69937	male	negative	40
69938	male	negative	40
69950	male	positive	26
69954	male	positive	40
69964	male	negative	40
65596	male	positive	70
65597	male	positive	66
65611	male	positive	43
65618	male	positive	46
65620	male	positive	20
65621	male	positive	49
65623	male	positive	73
65625	male	positive	32
65634	male	positive	20
65636	male	positive	26
65638	male	positive	40
65641	male	positive	20
65644	male	positive	55

65648	male	negative	27
65649	male	negative	53
65654	male	negative	56
65658	male	negative	37
65665	male	negative	22
65670	male	negative	49
65671	male	negative	62
65672	male	positive	70
65677	male	positive	20
65681	male	negative	61
65692	male	negative	26
65693	male	negative	20
65696	male	negative	54
65697	male	negative	57
65700	male	positive	34
65701	male	negative	20
65702	male	negative	57
65703	male	positive	20
65705	male	negative	32
65712	male	positive	20
65716	male	negative	33
65719	male	negative	33
65727	male	positive	39
65733	male	negative	39
65734	male	negative	35
65736	male	negative	39
65739	male	positive	43
65740	male	negative	43
65744	male	negative	39
65745	male	negative	39
65746	male	positive	56
65751	male	negative	48
65761	male	negative	20
65762	male	positive	36
